# Women’s and girls’ sexual empowerment differs by geographical context: a population-based validation study

**DOI:** 10.1186/s12905-025-03874-1

**Published:** 2025-07-05

**Authors:** Shannon N. Wood, Jessica L. Dozier, Celia Karp, Funmilola M. OlaOlorun, Elizabeth Omoluabi, Rosine Mosso, Pierre Z. Akilimali, Simon Peter Sebina Kibira, Frederick Makumbi, Georges Guiella, Peter Gichangi, Anoop Khanna, Sani Oumarou, Caroline Moreau

**Affiliations:** 1 Johns Hopkins Bloomberg School of Public Health, Baltimore, MD USA; 2 College of Medicine, University of Ibadan, Ibadan, Nigeria; 3 University of Western Cape, Cape Town, South Africa; 4 Ecole Nationale Superieure de Statistique et Appliquee d’Abidjan (ENSEA), Abidjan, Cote d’Ivoire South Africa; 5 Kinshasa School of Public Health, Kinshasa, Democratic Republic of Congo; 6 Makerere University School of Public Health, Kampala, Uganda; 7 Institut Supérieur des Sciences de la Population (ISSP/University Joseph Ki-Zerbo), Ouagadougou, Burkina Faso Burkina Faso; 8 International Centre for Reproductive Health-Kenya, Nairobi, Kenya; 9 Technical University of Mombasa, Mombasa, Kenya; 10 Department of Public Health and Primary Care, Faculty of Medicine and Health Sciences, Ghent University, Ghent, Belgium; 11 Indian Institute of Health Management Research, Maruti Nagar, Bangalore, India; 12 Institut National de la Statistique du Niger, Niamey, Nigeria; 13 Soins et Santé Primaire, CESP Centre for Research in Epidemiology and Population Health U1018, Inserm, Villejuif, F-94805 France; 14 Department of Population, Family and Reproductive Health, Johns Hopkins Bloomberg School of Public Health, 615 N. Wolfe Street W4511, Baltimore, MD 21205 USA

**Keywords:** Sexual health, Empowerment, Women, Girls

## Abstract

**Objective:**

To validate a sexual empowerment sub-scale of the Women’s and Girls’ Sexual and Reproductive Empowerment Index (WGE-SRH) across eight countries in sub-Saharan Africa and Asia, and compare sexual empowerment across these contexts.

**Methods:**

This study leverages cross-sectional, population-based survey data collected among women of reproductive age in Burkina Faso (*n* = 4,012), Côte d’Ivoire (*n* = 2,278); Kongo Central, Democratic Republic of the Congo (DRC (*n* = 1,097)); Kinshasa, DRC (*n* = 1,143); Rajasthan, India (*n* = 4,004); Kenya (*n* = 5,454); Kano, Nigeria (*n* = 780); Lagos, Nigeria (*n* = 804); Niger (*n* = 2,286); and Uganda (*n* = 2,228) to validate eight sexual empowerment measures via confirmatory factor analysis. Overall scores of the validated measures were descriptively examined across settings.

**Findings:**

Final models confirm the theoretical structure of the sexual empowerment measure, including two- dimensions: “existence of choice” and “exercise of choice”, each comprised of three items, with moderate internal consistency ranging from 0.59 to 0.69. Factor loadings, goodness-of-fit, and percent agreement varied in Rajasthan, India compared to sub-Saharan African settings. Marked variations were seen across sites in women’s perceptions of their partners’ responses to refusing sex, as well as their own confidence in voicing when and when not to have sex.

**Conclusion:**

This measure was developed in sub-Saharan Africa and works well for the population that it was designed to serve, however, cannot be extrapolated to other settings. To comprehensively capture the dynamic nature of sexual empowerment, future research is needed to examine measures across cultures and time. Validation across diverse settings opens avenues for holistic examination of positive sexual health, including factors that enhance women’s sexual empowerment and rights.

**Supplementary Information:**

The online version contains supplementary material available at10.1186/s12905-025-03874-1.

## Introduction

Women’s empowerment is at the fore of global development efforts given its substantial impact on women’s health and well-being [1–4]. A sizable body of evidence exists documenting relationships between women’s empowerment and a range of health behaviors and outcomes [5], including increased choice surrounding timing of sex [6], increased contraceptive use [7,8], decreased unintended pregnancy [9], and longer birth spacing [10]. Despite the multidimensional nature of empowerment [11–13], few studies have specifically examined sexual empowerment, a subdomain of empowerment, nor sought to understand its links to women’s sexual health.

Sexual health is an integral component of well-being and constitutes a wide range of physical, emotional, and social factors related to human sexuality [14]. It encompasses not only the absence of disease or dysfunction but also the presence of positive and respectful sexual experiences [14]. Empowering women in matters of sexual health is essential for women’s ability to control family planning and engage in safer sexual practices to prevent and manage sexually transmitted infections [7,8]. For example, increasing levels of sexual empowerment are associated with contraceptive use [7], which suggests improving women’s sexual empowerment may address more distal outcomes, such as unintended pregnancy.

Recent literature has defined sexual empowerment as a continuous and multidimensional process transitioning from existence of choices related to sexual activity to exercise of those choices, and, ultimately, achievement of choice [15]. The empowerment process begins with a woman’s internal and external constraints or motivations for choosing to have or avoid sex with a partner (existence of choice) [15]. It progresses to women’s confidence in exercising her choice, which influences her ability to engage in sexual negotiation with sexual partners and her capacity to make informed decisions about sex [15]. Given that women’s sexual empowerment is linked to gender norms, such as expectations that women should defer to men’s sexual desires and preferences, gender inequities may constrain women’s relative sexual power in heterosexual relations [16]. In situations where male partners hold disproportionate control over the timing and nature of sexual encounters, women’s capacity to negotiate sex may be limited [15], thus jeopardizing their ability to exercise control over sexual interactions and ultimately achieve their sexual goals [17].

Consistent measurement of sexual empowerment is important as positive sexual health is an outcome within itself, as well as a precursor to a range of health behaviors and outcomes given established links with contraceptive use and unintended pregnancy [7,8]. Women’s empowerment can vary substantially by setting, warranting a need to find cross-cultural commonalities to compare across settings [17]. To date, cross-cultural measures largely concentrate on proxies for sexual empowerment (i.e., norms towards wife beating) [18], or only capture limited aspects of sexual empowerment related to STI prevention [7,19–23], with little attention to gender and power dynamics that afford discussions and/or repercussions should sex be denied. Moreover, few sexual empowerment measures have focused on positive sexual health within a relationship [24]. More encompassing cross-cultural measures of sexual empowerment can create a more holistic picture of sexual health and progress towards meeting global development goals.

The Women’s and Girls’ Sexual and Reproductive Health Index (WGE-SRH), originally developed, refined, and validated in Ethiopia, Uganda, and Nigeria, aims to facilitate cross-cultural comparison of women’s empowerment to aid policymakers and programmatic efforts in tracking trends and progress toward population-level goals [17]. The WGE-SRH Index includes a promising sub-scale of sexual empowerment that can be tested in additional settings to understand whether current measures reflect women’s experiences of sexual empowerment across cultures. Thus, the purpose of the present study was to (1) validate the WGE-SRH sexual empowerment sub-scale across eight countries in sub-Saharan Africa and Asia and (2) compare women’s sexual empowerment across these diverse cultural contexts.

## Methods

### Overview of PMA

The present analyses use data from Performance Monitoring for Action (PMA), a research platform that administers annual population-based surveys on a range of topics related to sexual and reproductive health in eight countries in sub-Saharan Africa and Asia [25,26]. Surveys are administered by trained resident interviewers. Additional details can be found at pmadata.org [26].

The present analyses utilize cross-sectional Phase 1 PMA data collected in ten study sites from 2019 to 2020: (1) Burkina Faso; (2) Côte d’Ivoire; (3) Kongo Central, Democratic Republic of the Congo (DRC); (4) Kinshasa, DRC; (5) Kenya; (6) Kano, Nigeria; (7) Lagos, Nigeria; (8) Niger; (9) Rajasthan, India and (10) Uganda. Sites and phase were selected due to inclusion of the WGE-SRH Index within PMA surveys. Sites represent varying cultural and sexual practices both within and across contexts. Specifically, marriage less than age 18 is common in sites of Niger (76%) and Burkina Faso (51%) [27], both of which report some of the highest child marriage prevalences globally. Early sexual initiation (before the age of 15) was highest in Niger (67%) and the DRC (55%) [28]. Past-year intimate partner violence ranges substantially across sites, where it is estimated to be highest in the DRC (36%) and lowest in Burkina Faso (11%) [29].

All participants provided oral informed consent to participate; for minors, head of household informed consent was obtained per in country guidelines, as necessary. This study was approved by ethical review committees at Johns Hopkins School of Public Health and at each site in accordance with the Declaration of Helsinki and World Health Organization protocols.

### Measures

The primary measures of interest focus on sexual empowerment, as defined by the WGE-SRH framework [15,30] as the progression from the existence of choice through the exercise of choice to the achievement of choice; in this case, achievement of choice is volitional sex [15]. The sub-scale was piloted in three countries [17] and yielded eight items (four related to existence of choice and four related to exercise of choice).

In the present study, women indicated the extent they agreed or disagreed with each sexual empowerment item, with response values including 1-strongly disagree, 2-disagree, 3-neither agree nor disagree, 4-agree, 5-strongly agree (Table 1).

**Table 1:** WGE-SRH items

Existence of choice:
1) If I refuse sex with my husband/partner, he may stop supporting me
2) If I refuse sex with my husband/partner, he may force me to have sex
3) If I refuse sex with my husband/partner, he may physically hurt me
4) If I show my husband/partner that I want to have sex, he may consider me promiscuous
Exercise of choice:
1) I am confident I can tell my husband/partner when I want to have sex
2) I am able to decide when to have sex
3) If I do not want to have sex, I can tell my husband/partner
4) If I do not want to have sex, I am capable of avoiding it with my husband/partner.

### Analytic samples

**Fig. 1:**
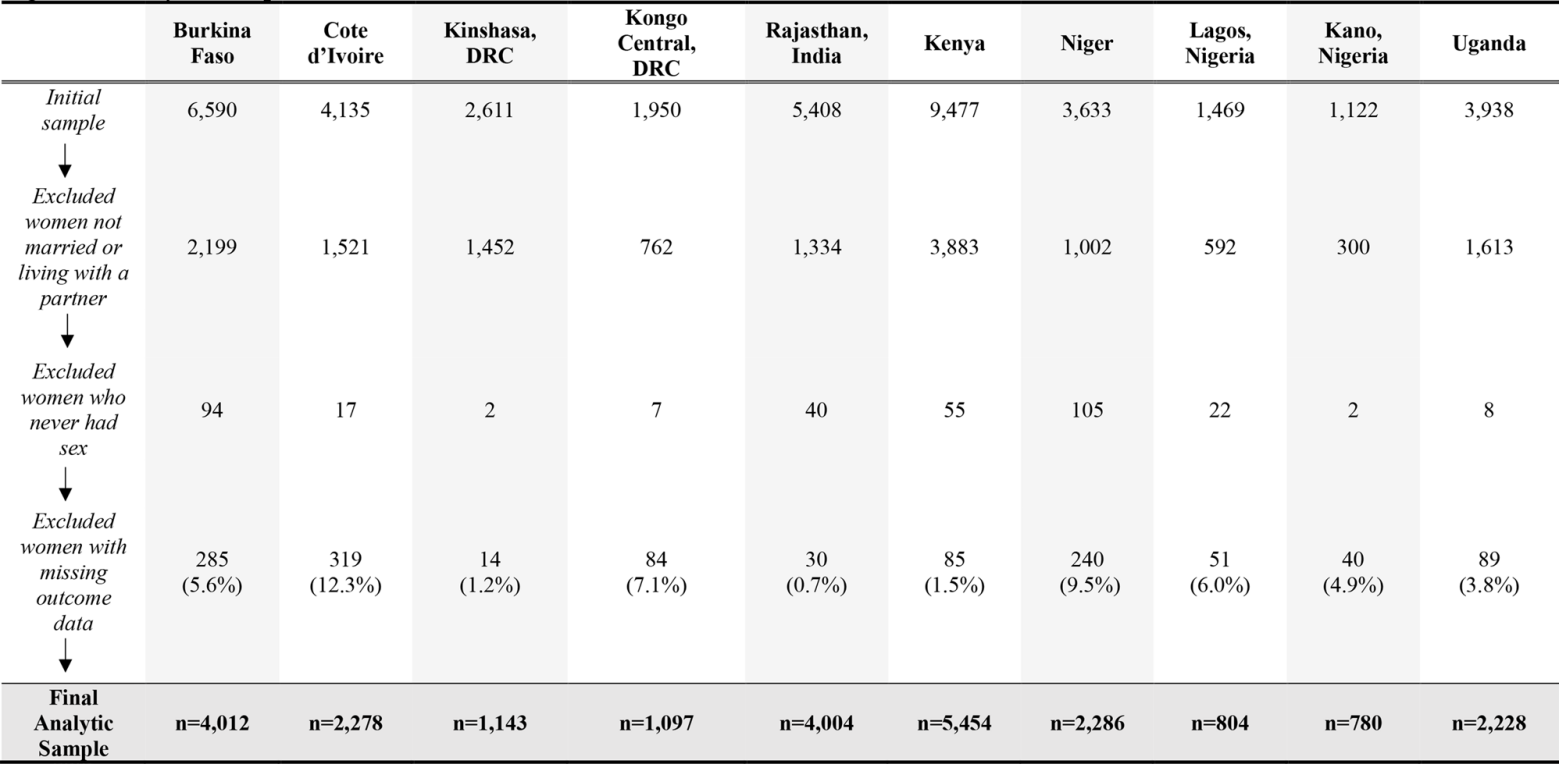
Analytic Sample Flowchart

Per site, analyses were restricted to women married or living with a partner as if married. Analytical samples were further restricted to women who had ever had sex as item wording emphasizes sexual negotiation and decision-making within ongoing partnerships, deeming sexual experience necessary for comprehension. Additionally, we utilized a complete case approach where participants missing WGE-SRH information for one or more items were excluded from the analytic sample (Fig. 1).

### Statistical analysis

Per site, weighted descriptive statistics first explored demographic characteristics of analytical samples and examined item distributions. In confirmatory factor analysis (CFA) models, WGE-SRH sexual empowerment item responses were treated continuously [1–5] and initial criterion for item retention included factor loadings (standardized coefficients) of ≥ 0.40 and p values < 0.05. By site, an initial two-factor model with all eight items was estimated via maximum likelihood estimation (Appendix 1). Items with factor loadings < 0.4 across multiple sites (specifically items: “*If I show my husband/partner that I want to have sex*,* he may consider me promiscuous”* and “*If I do not want to have sex*,*I am capable of avoiding it with my husband/partner”*) were considered for elimination. Then, the six-item reduced model was estimated for each site. Full and reduced models were compared by examining factor loading patterns, the proportion of variance accounted for, coefficient of determination, Akaike information criterion (AIC) and the Bayesian information criterion (BIC). Several additional indicators of model fit were examined, including: the Comparative Fit Index (CFI), the Tucker-Lewis Index (TLI), the Root Mean Square Error of Approximation (RMSEA), the Standardized Root Mean Square Residual (SRMR). Acceptable fit statistics followed criteria described by Schreiber et al. [31]. Cronbach’s alpha was used to assess internal consistency.

**Table 2:** Demographic characteristics of participants across study settings, weighted

	Burkina Faso (*n* = 4,012)	Cote d’Ivoire (*n* = 2,278)	Kinshasa, DRC (*n* = 1,143)	Kongo Central, DRC (*n* = 1,097)	Rajasthan, India (*n* = 4,004)	Kenya (*n* = 5,454)	Niger (*n* = 2,286)	Lagos, Nigeria (*n* = 804)	Kano, Nigeria (*n* = 780)	Uganda (*n* = 2,228)
**Characteristics**										
Residence										
Urban	693 (17.3)	1,214 (53.3)	1,143 (100)	1,097 (100)	993 (24.8)	1,580 (29.0)	327 (14.3)	804 (100)	233 (29.9)	569 (25.5)
Rural	3,319 (82.7)	1.064 (46.7)	-	-	3,011 (75.2)	3,874 (71.0)	1,959 (85.7)	-	547 (70.1)	1,659 (74.5)
Household Wealth Tertile										
Low	1,436 (35.8)	637 (28.0)	345 (30.2)	463 (42.2)	1,216 (30.4)	2,027 (37.2)	760 (33.2)	272 (33.8)	254 (32.5)	748 (33.6)
Middle	1,414 (35.3)	772 (33.9)	382 (33.4)	372 (34.0)	1,431 (35.7)	1,872 (34.3)	785 (34.3)	256 (31.8)	305 (39.1)	728 (32.7)
High	1,161 (29.9)	869 (38.2)	416 (36.4)	262 (23.9)	1,358 (33.9)	1,556 (28.5)	741 (32.4)	276 (34.4)	221 (28.4)	752 (33.7)
Age										
15–24	1,065 (26.6)	482 (21.2)	141 (12.3)	248 (22.7)	706 (17.6)	1,047 (19.2)	729 (31.9)	40 (4.9)	211 (27.1)	622 (27.9)
25–34	1,497 (37.3)	930 (40.8)	469 (41.1)	420 (38.3)	1,621 (40.5)	2,346 (43.0)	932 (40.8)	323 (40.1)	310 (39.7)	888 (39.9)
35–49	1,449 (36.1)	866 (38.0)	533 (46.6)	429 (39.1)	1,677 (41.9)	2,061 (37.8)	625 (27.3)	442 (55.0)	259 (33.2)	718 (32.2)
Parity										
0–2	1,184 (29.5)	840 (36.9)	401 (35.1)	351 (32.0)	2,034 (50.8)	1,946 (35.7)	595 (26.0)	334 (41.5)	170 (21.8)	709 (31.8)
3–4	1,075 (26.8)	638 (28.0)	409 (35.8)	328 (29.9)	1,339 (33.4)	1,794 (32.9)	622 (27.2)	331 (41.2)	155 (19.9)	572 (25.7)
5+	1,753 (43.7)	801 (35.1)	334 (29.2)	418 (38.1)	632 (15.8)	1,714 (31.4)	1,069 (46.8)	139 (17.3)	455 (58.4)	946 (42.5)
Education										
Never	2,838 (70.7)	1,224 (53.8)	5 (0.5)	150 (13.7)	1,592 (39.8)	333 (6.1)	1,732 (75.8)	19 (2.4)	466 (59.7)	183 (8.2)
Primary	689 (17.2)	606 (26.6)	128 (11.6)	362 (33.0)	901 (22.5)	2,808 (51.5)	324 (14.2)	97 (12.0)	142 (18.2)	1,348 (60.5)
Secondary+	484 (12.1)	448 (19.7)	1,010 (88.4)	585 (53.3)	1,511 (37.7)	2,314 (42.4)	230 (10.1)	688 (85.6)	173 (22.2)	697 (31.3)
Relationship										
Married	3612 (90.0)	1,415 (62.1)	724 (63.3)	456 (41.6)	3,998 (99.9)	4,921 (90.2)	2,272 (99.4)	730 (90.8)	777 (99.6)	973 (43.7)
Living with Partner	400 (10.0)	863 (37.9)	419 (36.7)	641 (58.4)	6 (0.1)	533 (9.8)	14 (0.6)	74 (9.2)	3 (0.4)	1,255 (56.3)
Polygynous Relationship	1,794 (44.7)	570 (25.0)	86 (7.5)	147 (13.4)	107 (2.7)	794 (14.6)	847 (37.1)	92 (11.4)	363 (46.5)	596 (26.7)

Analyses limited to women currently married or living with a partner as if married and ever sexually active

Site-specific summary scores for each latent sexual empowerment domain (i.e., existence of choice, exercise of choice) were computed by averaging scores from retained items. Overall site-specific sexual empowerment scores were computed by averaging existence of choice and exercise of choice scores to ensure equal weighting across domains. Higher scores (range: 1–5) reflected higher levels of sexual empowerment.

Statistical significance was set a priori at*p* < 0.05. Analyses were conducted in STATA version 16 (StataCorp. 2019. Stata: Release 16. Statistical Software. College Station, TX: StataCorp LLC) and accounted for complex survey design.

## Results

### Sample characteristics

Across all sites, except Côte d’Ivoire, most ever sexually active, married or cohabitating women lived in rural communities (Table 2); notably, the samples for Lagos, Kinshasa, DRC and Kongo Central, DRC include only urban communities. Approximately 40% of women across sites were aged 25–34, and most had three or more children in all sites except Rajasthan, India, where more than half (50.8%) of participants had less than two children. Highest level of schooling varied widely by site. Polygynous unions were most common in Kano, Nigeria (46.5%) and Burkina Faso (44.7%), and least common in Kinshasa, DRC (7.5%) and Rajasthan, India (2.7%).

### Existence of choice

**Fig. 2:**
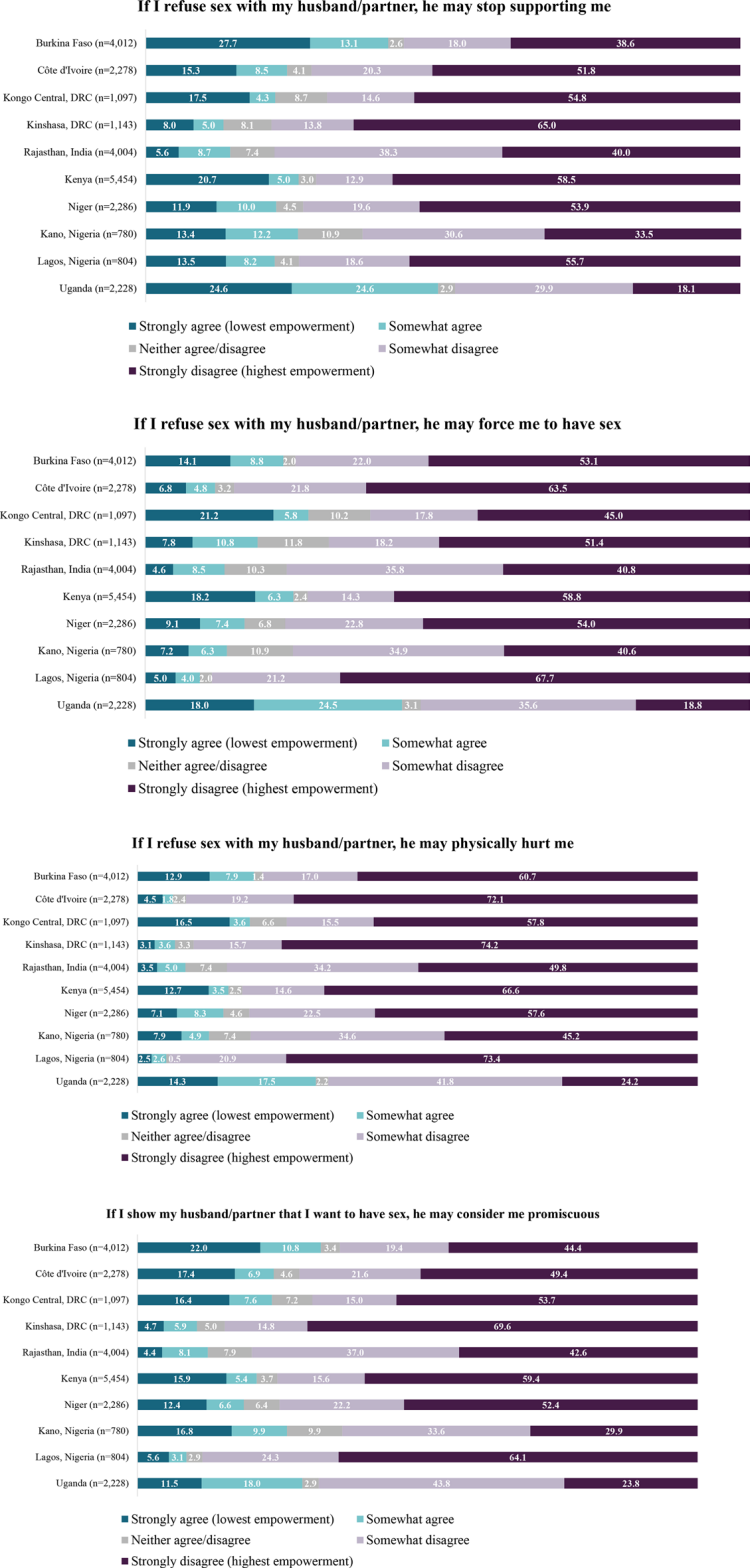
Distribution of agreement for WGE-SRH existence of choice items.*Existence of choice items are reverse-coded*

Women’s internal and external constraints and motivations for choosing to have or avoid sex (existence of choice) varied across sites and items (Fig. 2). Between 5.6% of women in Rajasthan, India and 27.7% of women in Burkina Faso strongly agreed (score = 1) that their partner would stop supporting them if they refused sex. Less than one in four women across sites indicated strong agreement that their partner would force them to have sex if they refused (range: 4.6% in Rajasthan, India to 21.2% in Kongo Central, DRC). Fewer women strongly agreed that their partner would physically hurt them if they refused sex (range: 2.5% in Lagos, Nigeria to 16.5% in Kongo Central, DRC). Strong agreement with the item, “*If I show my partner that I want to have sex*,* he may consider me promiscuous*,*”* ranged from 4.4% in Rajasthan, India to 22.0% in Burkina Faso.

### Exercise of choice

**Fig. 3:**
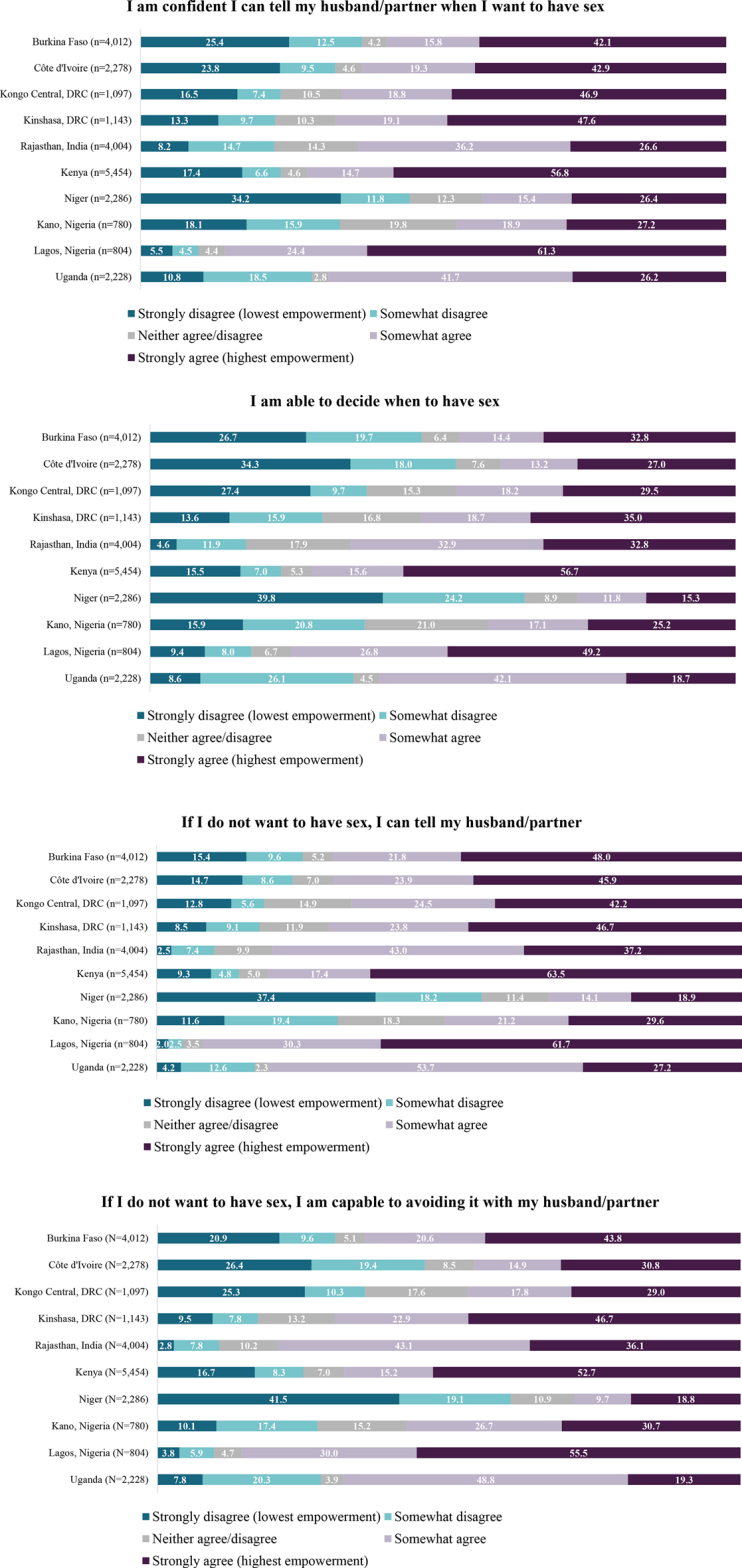
Distribution of agreement for WGE-SRH exercise of choice items

The distribution of agreement for exercise of choice items also varied by site (Fig. 3). Across sites, more than one in four women indicated high confidence in their ability to tell their partner when they wanted sex (ranging from 26.2% in Uganda to 61.3% in Lagos, Nigeria). The greatest proportion of women responded affirmatively that they could decide when to have sex; strong agreement for this item (“*I am able to decide when to have sex”)* was lowest in Niger (15.3%).

### Confirmatory factor analysis

Two items were dropped in final models due to factor loadings < 0.4 across multiple sites. Specifically, factor loadings were < 0.4 in four sites for the item “*If I show my husband/partner that I want to have sex*,* he may consider me promiscuous”* (standardized coefficients: 0.26 in Kongo Central, DRC to 0.70 in Rajasthan, India), and < 0.4 in three sites for*“If I do not want to have sex*,* I am capable of avoiding it with my husband/partner”* (standardized coefficients: 0.32 in Kongo Central, DRC to 0.79 in Rajasthan, India; (Supplemental Table1; Supplemental Fig. 1).

**Fig. 4:**
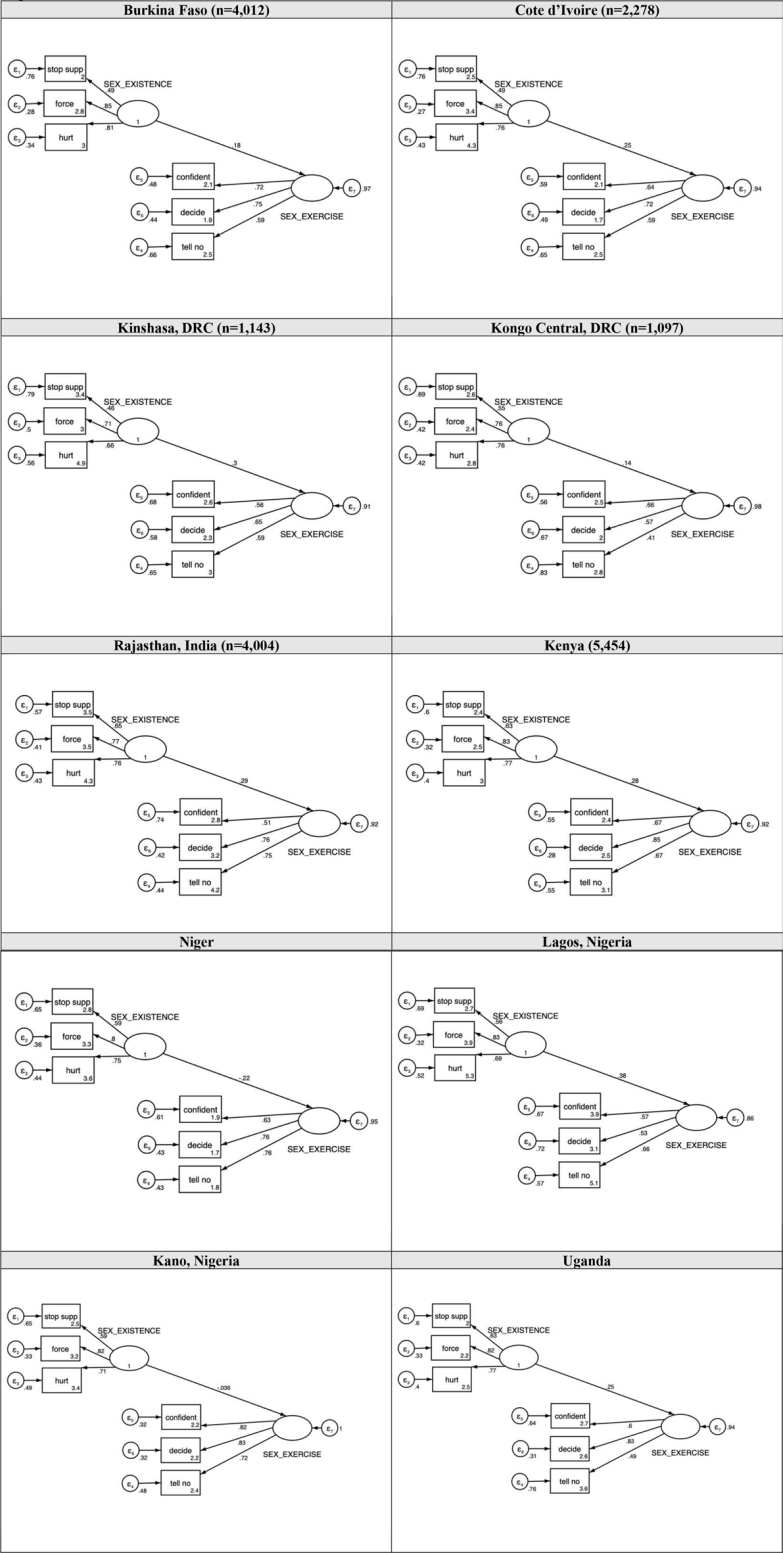
Final path models. Notes: Sex_Existence = Existence of Choice; Sex_Exercise = Exercise of Choice; Stop supp = If I refuse sex with my husband/partner, he may stop supporting me; Force = If I refuse sex with my husband/partner, he may force me to have sex; Hurt = If I refuse sex with my husband/partner, he may physically hurt me; Confident = I am confident I can tell my husband/partner when I want to have sex; Decide = I am able to decide when to have sex; Tell no = If I do not want to have sex, I can tell my husband/partner

The final CFA models supported a two-factor scale consistent with the hypothesized WGE-SRH sexual empowerment construct (Table 3; Fig. 4). Across sites, items demonstrated strong factor loadings: “*If I refuse sex with my husband/partner*,* he may stop supporting me”* (standardized coefficients: 0.46 in Kinshasa, DRC to 0.65 in Rajasthan, India); “*If I refuse sex with my husband/partner*,* he may force me to have sex”* (standardized coefficients: 0.71 in Kinshasa, DRC to 0.85 in Burkina Faso and 0.85 in Cote d’Ivoire ); “*If I refuse sex with my husband/partner*,* he may physically hurt me”* (standardized coefficients: 0.66 in Kinshasa, DRC to 0.81 in Burkina Faso); “*I am confident I can tell my husband/partner when I want to have sex”* (standardized coefficients: 0.51 in Rajasthan, India to 0.82 in Kano, Nigeria); “*I am able to decide when to have sex”* (standardized coefficients: 0.53 in Lagos, Nigeria to 0.85 in Kenya); “*If I do not want to have sex*,* I can tell my husband/partner”* (standardized coefficients: 0.41 in Kongo Central, DRC to 0.75 in Rajasthan, India).

**Table 3:** Confirmatory factor analysis (CFA), final results for sexual empowerment by site

	Burkina Faso (*n* = 4,012)	Cote d’Ivoire (*n* = 2,278)	Kinshasa, DRC (*n* = 1,143)	Kongo Central, DRC (*n* = 1,097)	Rajasthan, India (*n* = 4,004)	Kenya (*n* = 5,454)	Niger (*n* = 2,286)	Lagos, Nigeria (*n* = 804)	Kano, Nigeria (*n* = 780)	Uganda (*n* = 2,228)
CFI	0.998	0.996	0.988	0.989	0.970	0.994	0.990	1.00	0.997	0.998
TLI	0.997	0.993	0.978	0.979	0.944	0.989	0.982	1.00	0.995	0.996
RMSEA	0.018	0.025	0.034	0.036	0.074	0.037	0.043	< 0.001	0.026	0.020
p(RMSEA)	1.00	0.999	0.890	0.851	< 0.001	0.996	0.797	0.999	0.920	1.00
SRMR	0.016	0.018	0.024	0.028	0.037	0.023	0.028	0.012	0.029	0.017
AIC	82,532	45,875	21,819	23,029	67,146	109,993	45,366	13,728	14,504	42,237
BIC	82,652	45,984	21,915	23,124	67,266	110,119	45,475	13,817	14,593	42,345
CD	0.830	0.815	0.681	0.762	0.783	0.815	0.784	0.787	0.781	0.811
Alpha(α)	0.67	0.66	0.61	0.59	0.68	0.73	0.69	0.65	0.66	0.68

Desirable indices from Scheiber el al., 2006: Comparative Fit Index, CFI > 0.96; Tucker-Lewis Index, TLI > 0.95; Root mean square error of approximation, RMSEA < 0.06; Standardized Root Mean Residual, SRMR < 0.08; Coefficient of Determination (CD). Internal reliability Cronbach’s alpha(α):*≥*0.70

CFA confirmed goodness of fit across all sites [31]. However, in Rajasthan, results for TLI and RMSEA metrics were within one-tenth of desirable values. All other metrics were suitable across sites. The majority of sites reported moderate internal consistency: Kongo Central, DRC (α = 0.59), Kinshasa, DRC (α = 0.61), Lagos, Nigeria (α = 0.65), Kano, Nigeria (α = 0.66), Cote d’Ivoire (α = 0.66), Burkina Faso (α = 0.67), Rajasthan, India (α = 0.68), Uganda (α = 0.68), and Niger (α = 0.69).

### Overall sexual empowerment across sites

**Fig. 5:**
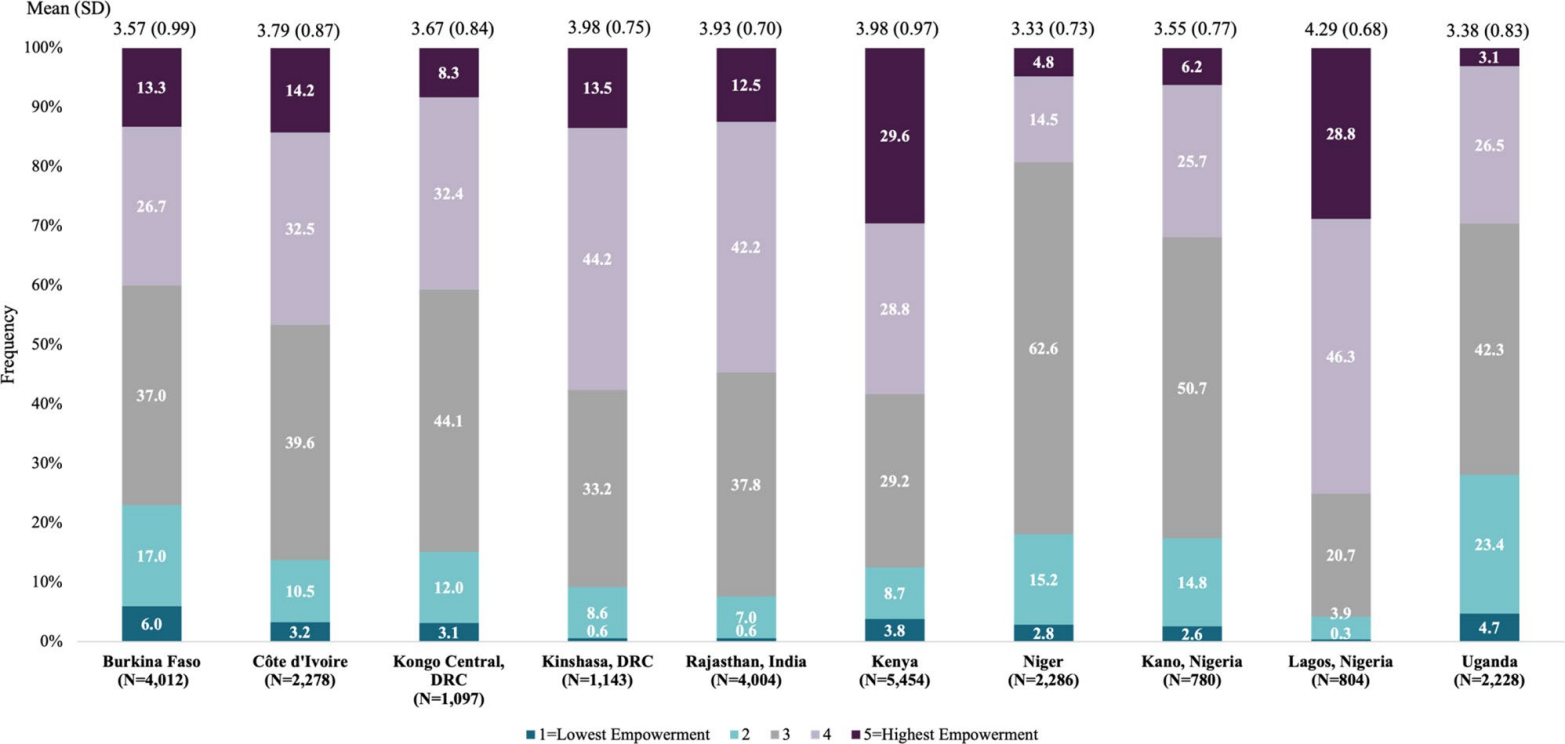
Distribution of overall sexual empowerment scores by site

The proportion of women with the highest empowerment scale scores ranged from 4.8% in Niger to 29.6% in Kenya (Fig. 5). Fewer than one in ten women in Kongo Central, DRC (8.3%), Niger (4.8%), Kano, Nigeria (6.2%), and Uganda (3.1%) reported highest empowerment. The proportion of women with the lowest scores ranged from < 1% in Lagos, Nigeria to 6.0% in Burkina Faso.

## Discussion

This study is the first to examine the psychometric properties and factor structure of the sexual empowerment sub-scale of the WGE-SRH Index across ten culturally diverse settings. Overall, results suggest the scale is a reliable tool for understanding how women frame and act on sexual decisions across diverse cultures in sub-Saharan Africa. After dropping two items, psychometric properties were strong in most sites, except Rajasthan, India, where item fit remained slightly below desired thresholds.

Findings confirm a two-factor structure of sexual empowerment (existence of choice and exercise of choice), similar to that originally identified by Moreau and colleagues [17]. Notably, however, some items performed differently across contexts. In Rajasthan, India, in particular, full models showed that the item,*“If I show my husband/partner that I want to have sex*,* he may consider me promiscuous*” loaded strongly onto the existence of choice domain, while the item, “*If I do not want to have sex*,* I am capable of avoiding it with my husband/partner”* loaded strongly onto exercise of choice. In contrast, factor loadings for these two items showed considerable variability in sub-Saharan African settings, with the promiscuity item underperforming in the majority of contexts. Similarly, the “avoid” item did not meet desired thresholds in several sites. As such, both items were dropped from final models to ensure consistency across sub-Saharan African sites, however, these items may still have relevance and be considered as critical aspects of sexual empowerment within the context of research in Asia, where commonality of extramarital relationships and implications of promiscuity may differ [32,33]. Combining the sexual existence of choice and sexual exercise of choice domains into a single measure resulted in a multidimensional index of sexual empowerment with moderate to good internal reliability (alpha range 0.59 in Kongo Central, DRC to 0.69 in Niger), along with evidence of goodness of fit across sites, withstanding Rajasthan, India.

Understanding cross-site differences in existence vs. exercise of choice is relevant for the implementation of interventions and programs seeking to counteract harmful gender norms. Marked variations were seen across sites in women’s perceptions of their partners’ responses to refusing sex, as well as their own confidence in voicing when and when not to have sex. Niger, specifically, stood out as a site with overall low exercise of choice; however, this setting was comparable to other sites for existence of choice items. Such contrast between existence and exercise of choice may be indicative of where a population lies on the empowerment pathway [15]. Of note, Niger has the highest prevalence of child marriage globally [27]. While many contexts have overcome gender and power barriers related to existence of choice surrounding sex, many women still may not feel comfortable exercising this choice. In such contexts, programs and interventions should focus on supporting women’s ability to communicate and negotiate their sexual choices, rather than normative factors prohibiting women’s sexual needs in the first place. Community dialogues, such as those implemented for intimate partner violence, may be useful to support communication and negotiation, while concurrently working with men and the broader community [34–36]. As opposed to contraceptive empowerment, where women can exercise their choice to use contraception via covert contraceptive use without first having the existence of choice [30,37], sex requires an interaction between sexual partners and, therefore, both existence and exercise of choice must co-exist for women to achieve volitional sex. These results point to the importance of disentangling the domains of existence vs. exercise of choice for sexual empowerment.

Identifying within-site variation is also necessary to determine where empowerment interventions are needed or may have fallen short. Some items were particularly polarizing within contexts—specifically, the “promiscuous” item had the majority responses concentrating in strongly agree or strongly disagree categories, with few women indicating neutral empowerment (i.e., neither agreeing nor disagreeing). These polarized responses persisted for exercise of choice items across contexts and were particularly pronounced for items focusing on women’s desire to have sex rather than not to have sex. Such results highlight the sexual double standards between men and women reported in previous studies [15,38–40] and speak to the undervaluing of women’s sexual pleasure as a key component of their sexual health [41].

These cross-cultural findings further elucidate that sexual empowerment is largely constrained—when examining our overall sexual empowerment scores, less than half of the sites reported the majority of women with high or highest empowerment scores (Lagos, Nigeria: 74.5%, Kenya: 58.4%, Kinshasa, DRC: 57.7%, and Rajasthan, India: 54.7%). In sites where existence and exercise of choice were in opposition (i.e., Niger, Kano, Nigeria, and Kongo Central, DRC), overall empowerment concentrated in the neutral category. Notably, these lower empowerment sites likely represent different stages of gender norms programs (i.e., focus on existence of choice rather than exercise of choice) and should continue to be examined.

This study has several strengths, including distinguishing between sexual existence and exercise of choice and its use of ten population-based samples to validate a sexual empowerment sub-scale and compare sexual empowerment levels across sites. Nonetheless, findings should be considered in light of some limitations. Specifically, item wording pertains to a woman’s current husband/partner, precluding understanding of how these items apply within concurrent partner relationships. Women may feel empowered sexually with some partners, but not with others. Within-site variation in sexual empowerment was not examined to afford fuller exploration of sexual empowerment across populations, however, given cultural heterogeneity in practices within many study sites, this should be examined in future studies. Reasons for sexual empowerment within and across sites, as well as an understanding of who is empowered, as additionally needed. Lastly, it is noted that only one small site within Asia was included in this study (Rajasthan, India) and is not generalizable to the whole of Asia nor the whole of India.

## Conclusion

Despite these limitations, results suggest that the WGE-SRH scale is a reliable tool for understanding how women frame and act on sexual decisions across diverse cultures in sub-Saharan Africa. Further, it provides an opportunity to identify factors that enhance women’s sexual empowerment and the contribution to sexual empowerment towards improving various aspects of women’s health and well-being. Additional data are needed to understand how partner and couple characteristics impact sexual relationships, power dynamics, and sexual goal setting. Moreover, longitudinal designs are needed to examine the progression from existence of choice to achievement of choice (volitional sex) that may change across different stages of the life course, and to examine how sexual empowerment relates to SRH outcomes.

## Electronic supplementary material

Below is the link to the electronic supplementary material.

Supplementary Material 1

## Data Availability

Data are publicly available at pmadata.org.

## Abbreviations

AIC: Akaike Information Criterion
BIC: Bayesian Information Criterion
CFA: Confirmatory Factor Analysis
CFI: Comparative Fit Index
DRC: Democratic Republic of the Congo
PMA: Performance Monitoring for Action
RMSEA: Root Mean Square Error of Approximation
SRMR: Standardized Root Mean Square Residual
TLI: Tucker-Lewis Index
WGE-SRH: Women’s and Girls’ Empowerment in Sexual and Reproductive Health
