# Supplementary material for: Women’s and girls’ sexual empowerment differs by geographical context: a population-based validation study
Source: BMC Womens Health. 2025 Jul 5;25:331. doi: 10.1186/s12905-025-03874-1 (PMC12228322; doi:10.1186/s12905-025-03874-1)
Supplement: Supplementary file 1 — Supplementary Material 1 [file 12905_2025_3874_MOESM1_ESM.docx]

**Supplemental Table 1. Confirmatory Factor Analysis (CFA), initial results for sexual empowerment by site (all 8 items retained)**

|  | **Burkina**  **Faso**  **(n=4,012)** | **Cote d’Ivoire**  **(n=2,278)** | **Kinshasa, DRC**  **(n=1,143)** | **Kongo**  **Central,**  **DRC**  **(n=1,097)** | **Rajasthan,**  **India**  **(n=4,004)** | **Kenya**  **(n=5,454)** | **Niger**  **(n=2,286)** | **Lagos, Nigeria**  **(n=804)** | **Kano, Nigeria**  **(n=780)** | **Uganda**  **(n=2,228)** |
| --- | --- | --- | --- | --- | --- | --- | --- | --- | --- | --- |
| CFI | 0.915 | 0.895 | 0.863 | 0.933 | 0.957 | 0.950 | 0.982 | 0.958 | 0.949 | 0.916 |
| TLI | 0.874 | 0.846 | 0.798 | 0.902 | 0.937 | 0.927 | 0.974 | 0.938 | 0.925 | 0.876 |
| RMSEA | 0.093 | 0.095 | 0.088 | 0.062 | 0.078 | 0.080 | 0.044 | 0.062 | 0.082 | 0.093 |
| p(RMSEA) | <0.001 | <0.001 | <0.001 | 0.047 | <0.001 | <0.001 | 0.854 | 0.082 | <0.001 | <0.001 |
| SRMR | 0.068 | 0.072 | 0.066 | 0.045 | 0.040 | 0.048 | 0.032 | 0.046 | 0.054 | 0.057 |
| AIC | 111497 | 62707 | 29174 | 31024 | 85824 | 147267 | 60474 | 18094 | 19444 | 56145 |
| BIC | 111655 | 62851 | 29300 | 31149 | 85981 | 147432 | 60617 | 18211 | 19561 | 56288 |
| CD | 0.830 | 0.809 | 0.690 | 0.765 | 0.822 | 0.821 | 0.797 | 0.786 | 0.796 | 0.819 |
| Alpha(α) | 0.71 | 0.68 | 0.64 | 0.60 | 0.76 | 0.77 | 0.74 | 0.72 | 0.59 | 0.73 |

*Notes: Desirable indices from Scheiber el al., 2006: Comparative Fit Index, CFI > 0.96; Tucker-Lewis Index, TLI > 0.95; Root mean square error of approximation, RMSEA < 0.06; Standardized Root Mean Residual, SRMR < 0.08; Coefficient of Determination (CD). Internal reliability Alpha(α): >0.70.*

**Supplemental Figure 1. Full Path Models**

| **Burkina Faso** | **Cote d’Ivoire** |
| --- | --- |
| 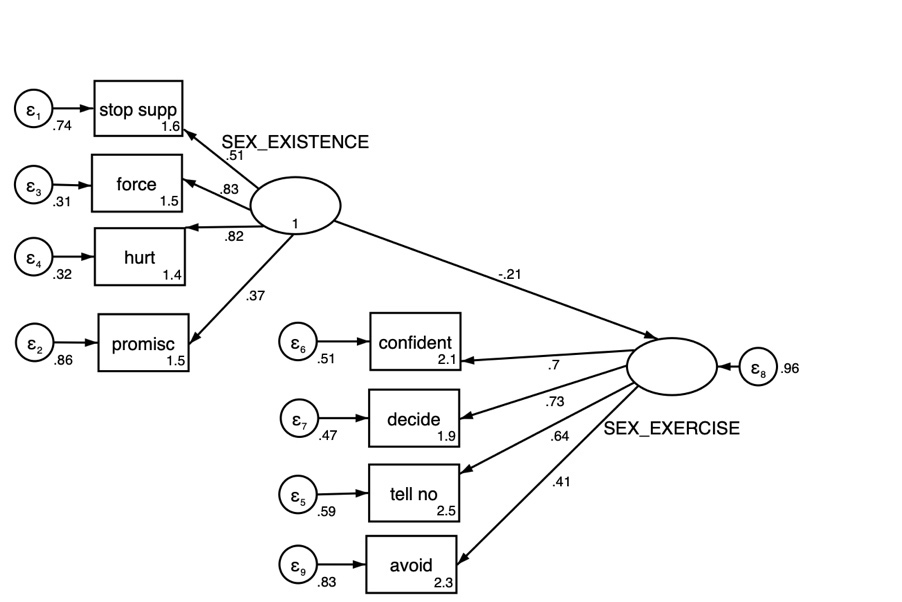 | **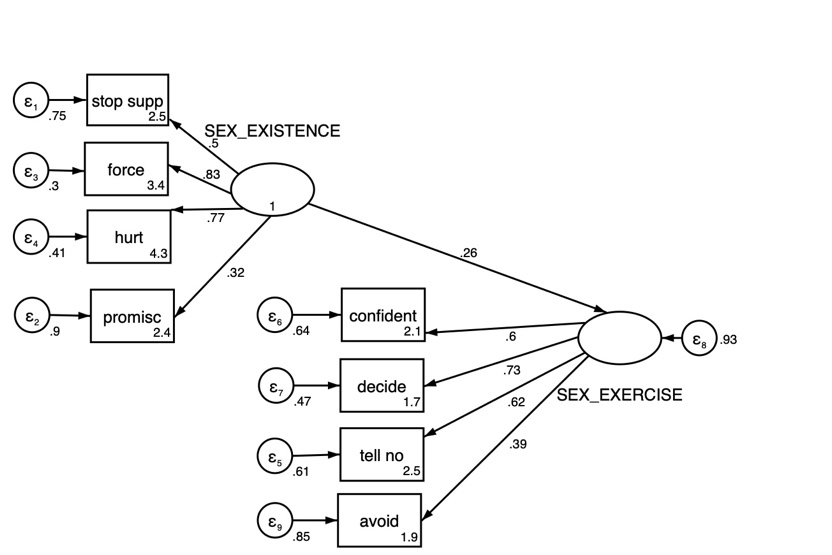** |
| **Kinshasa, DRC** | **Kongo Central, DRC** |
| **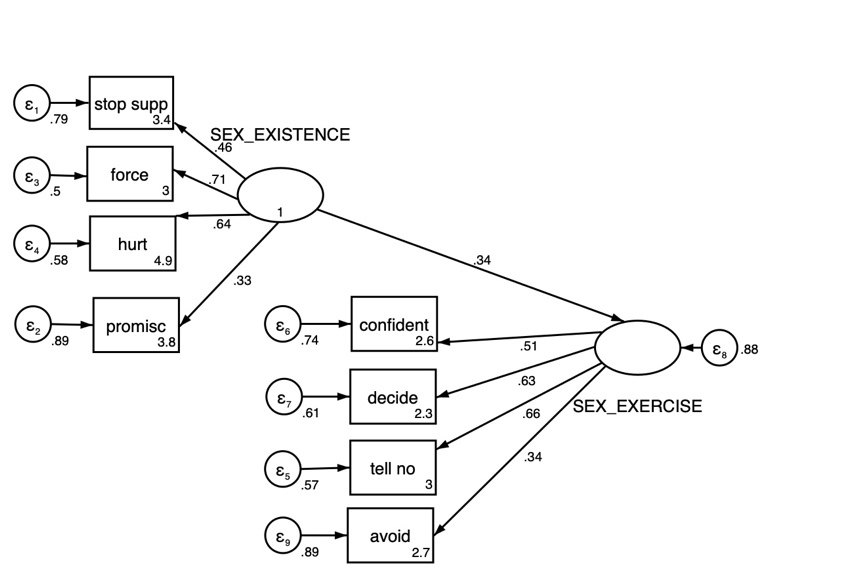** | **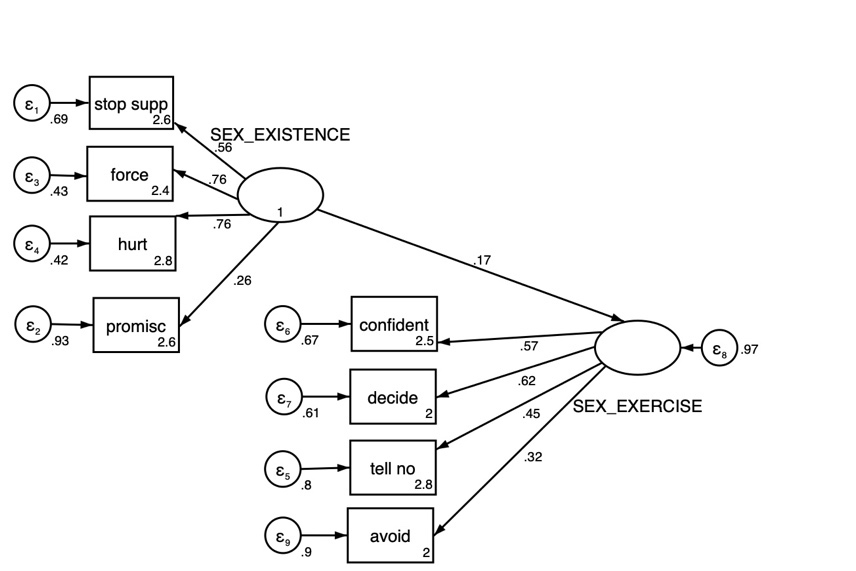** |
| **Rajasthan, India** | **Kenya** |
| **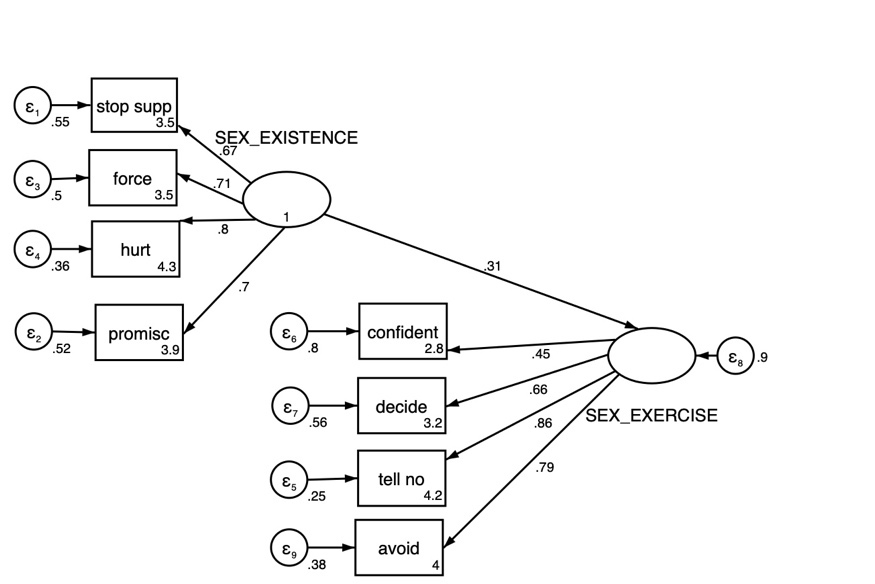** | **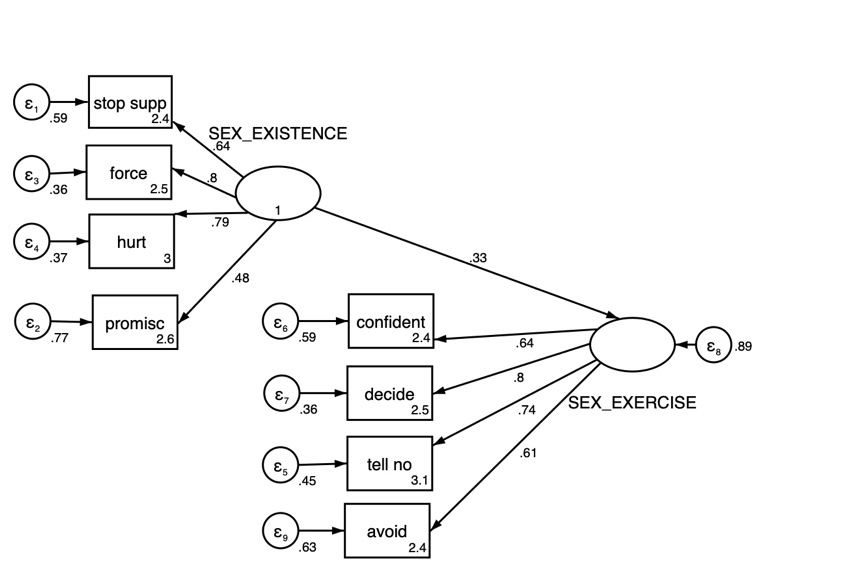** |
| **Niger** | **Lagos, Nigeria** |
| **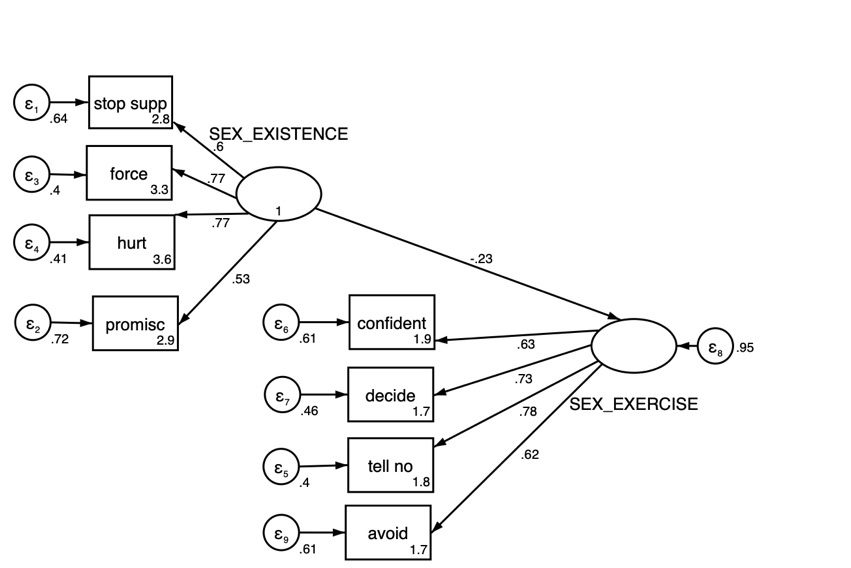** | **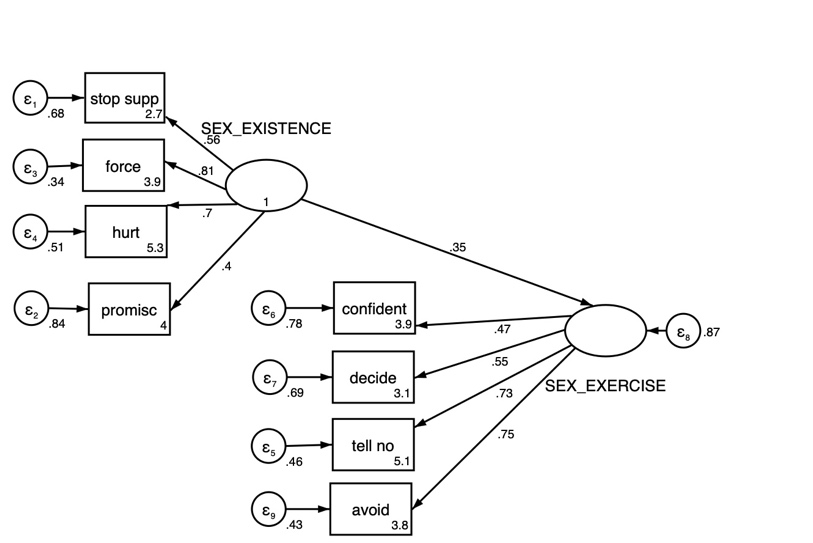** |
| **Kano, Nigeria** | **Uganda** |
| **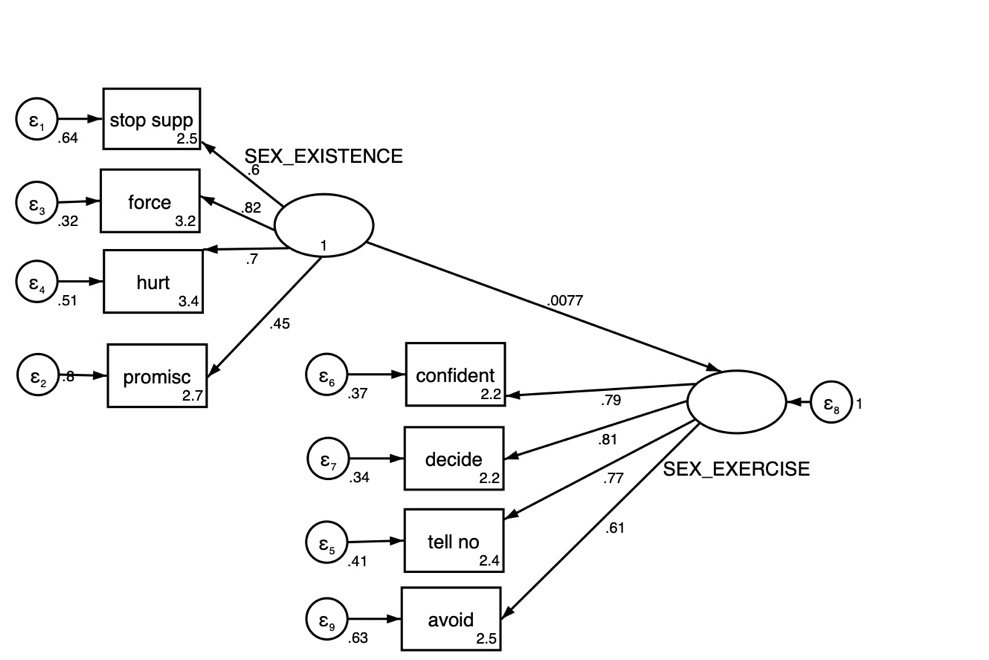** | **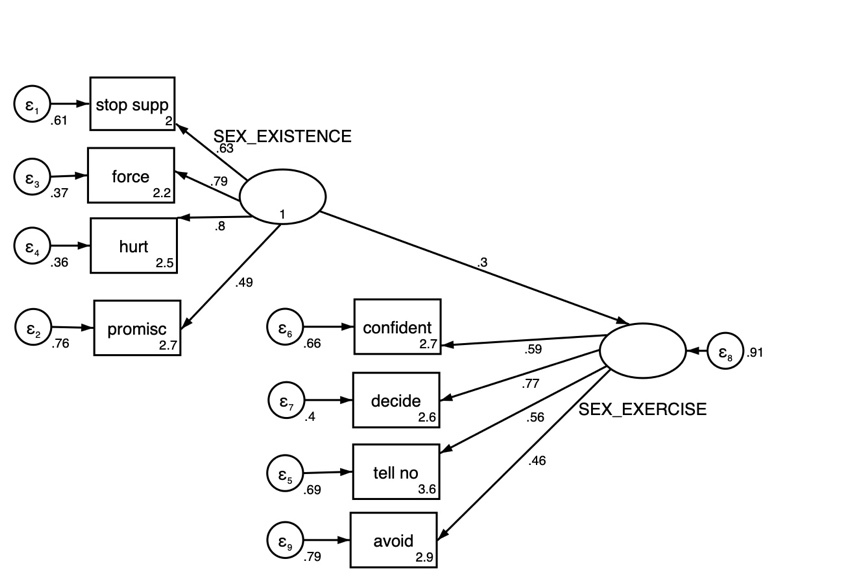** |

*Notes: Sex_Existence=Existence of Choice; Sex_Exercise=Exercise of Choice; Stop supp=If I refuse sex with my husband/partner, he may stop supporting me; Force= If I refuse sex with my husband/partner, he may force me to have sex; Hurt= If I refuse sex with my husband/partner, he may physically hurt me; Confident= I am confident I can tell my husband/partner when I want to have sex; Decide= I am able to decide when to have sex; Tell no= If I do not want to have sex, I can tell my husband/partner; promiscuous=If I show my husband/partner that I want to have sex, he may consider me promiscuous; avoid=If I do not want to have sex, I am capable of avoiding it with my husband/partner*
